# Perceptions of climate change and its impact on human health: an integrated quantitative and qualitative approach

**DOI:** 10.3402/gha.v7.23025

**Published:** 2014-12-08

**Authors:** Do Thi Thanh Toan, Vu Duy Kien, Kim Bao Giang, Hoang Van Minh, Pamela Wright

**Affiliations:** 1 Department of Biostatistics and Medical Informatics, Institute of Training for Preventive Medicine and Public Health, Hanoi Medical University, Hanoi, Vietnam; 2 Center for Health System Research, Hanoi Medical University, Hanoi, Vietnam; 3 Department of Health Promotion, Institute of Training for Preventive Medicine and Public Health, Hanoi Medical University, Hanoi, Vietnam; 4 Department of Health Economic, Institute of Training for Preventive Medicine and Public Health, Hanoi Medical University, Hanoi, Vietnam; 5 The Medical Committee Netherlands – Vietnam, Hanoi, Vietnam

**Keywords:** climate change, perception, health, Hanoi, Vietnam

## Abstract

**Background:**

The World Health Organization emphasized that climate change is a significant and emerging threat to public health, especially in lower income populations and tropical/subtropical countries. However, people in Asia and Africa were the least likely to perceive global warming as a threat. In Vietnam, little research has been conducted concerning the perceptions of effects of climate change on human health.

**Objective:**

The aim of this study was to explore the perceptions on climate change and its impact on human health among people in Hanoi.

**Design:**

We applied a combined quantitative and qualitative approach to study perceptions on climate change among people in Hanoi. A total of 1,444 people were recruited, including 754 people living in non-slum areas and 690 people living in slum areas of Hanoi. A structured questionnaire was used to collect quantitative data on their perceptions. In a parallel qualitative study, two focus group discussions and 12 in-depth interviews (IDs) were carried out involving 24 people from both slum and non-slum areas.

**Results:**

The majority of the respondents in the study had heard about climate change and its impact on human health (79.3 and 70.1% in non-slum and slum areas, respectively). About one third of the respondents reported that members of their family had experienced illness in the recent summer and winter compared to the same seasons 5 years ago. The most common symptoms reported during hot weather were headaches, fatigue, and dizziness; hypertension and other cardiovascular diseases were also reported. During cold weather, people reported experiencing cough, fever, and influenza, as well as pneumonia and emerging infectious diseases such as dengue and Japanese encephalitis.

**Conclusions:**

The observed high level of awareness on the links between climate change and human health may help to increase the success of the National Prevention Program on Climate Change. Moreover, understanding the concerns of the people may help policy makers to develop and implement effective and sustainable adaptation measures for Hanoi City as well as for Vietnam as a whole.

Climate change has already been observed globally, including increases in air and water temperatures, reduced number of frost days, increased frequency and intensity of heavy downpours, a rise in sea level, and reductions in snow cover, glaciers, permafrost, and sea ice (1). In 1990, the Intergovernmental Panel on Climate Change concluded that there was broad international consensus that climate change is human induced (2). However, in a study conducted by a Gallup Poll in 128 countries, still one third of residents from Africa, parts of Asia and the Middle East, and a few countries from the former Soviet Union did not agree that climate change is a result of human activities (3).

The link between population health and climate change has been demonstrated by scientists who stated that climate change poses a wide range of risks to population health. As noted by the World Health Organization, climate change is not just a threat to biological systems and the environment but a ‘significant and emerging threat to public health’, especially in lower income populations and tropical/subtropical countries (2, 4). Protecting health from the impacts of climate change is recognized as one of the defining challenges in this century (1). Perceptions on the effects of climate change on health have been studied among community members in developed (5–10) and developing countries (11–14). Haque et al. (13) found in Bangladesh that most local perceptions on climate change were consistent with the scientific evidence regarding the vulnerability of that country to climate change. The people perceived that changes in heat, cold, and rainfall had occurred over the last 5–10 years and linked these problems to identify future threats to themselves and their families and livelihoods (13).

Vietnam is one of the developing countries most affected by climate change (15, 16). The country suffers from many kinds of natural disasters that affect an estimated 1 million Vietnamese annually. The impact of climate change on individuals varies according to many factors like age, occupation, gender, education, and economic status (4, 7, 17, 18). In addition, factors that influence concern about climate change include past experiences of the impact of flooding on livelihood and observation of changes in the climate (19). Understanding the complex links between climate change and human health as well as understanding peoples’ concerns will assist policy makers to develop communication strategies to engage communities in each location most effectively to deal with the consequences of climate change. Such communication is increasingly a priority for Hanoi City, because in recent years, climate change has affected the city with extreme consequences (17).

Nonetheless, to the best of our knowledge, very little research has been conducted in Vietnam about peoples’ perceptions of the effect of climate change on human health. Therefore, the aim of this study was to explore Hanoian's perceptions of climate change and its effect on their health. We also looked for relationships between peoples’ perceptions and a set of socioeconomic characteristics that might influence their knowledge and experiences. The results of this study could be used to inform decision makers in Hanoi responsible for developing or adapting programs of action to cope with climate change.

## Methods

### Study setting

Hanoi, the capital of Vietnam, comprises 29 districts, 10 of which are urban and 19 rural. As of 2009, Hanoi's population was estimated at 6.5 million, of which 2.6 million (41%) lived in urban districts (20). The study was carried out in four urban districts in Hanoi, namely Ba Dinh, Hoan Kiem, Hai Ba Trung, and Dong Da districts, because they included a number of slum areas. We defined the slum areas according to the United Nations definition, as areas where people live in temporary houses, insecure locations, narrow spaces, or polluted environmental conditions.

### Study design and participants

This was a population-based cross-sectional study conducted from December 2012 to March 2013, applying a mixed-methods research design, quantitative and qualitative (21, 22). To obtain a wider range of household perceptions from a large number of households, a quantitative survey was conducted in 30 slum areas (randomly selected from the list of 84 slum areas) and 30 matched, nearby non-slum areas in the four urban districts. In each slum or non-slum area, about 20 households were selected; we applied a door-to-door approach after randomly selecting the first house. In each household, we selected one individual among the household members between 15 and 60 years of age by chance, and a second individual above 60 years old, if available. The non-response rate was only 2–3%, and quite similar in both slum and non-slum areas. Finally, 1,444 people were invited to participate, including 754 people in non-slum areas and 690 people in slum areas. We included a qualitative study to gain deeper insights into local perceptions and experiences of people in Hanoi. Two focus group discussions (FGDs) and 12 key informant interviews were conducted. The participants were selected purposively from both slum and non-slum areas in Hanoi. They included 16 community members, four local political leaders and four district health staff.

### Data collection

In the quantitative study, face-to-face interviews using structured questionnaires were conducted by trained medical students from Hanoi Medical University. The interviewers were supervised by senior staff from the Center for Health System Research, Hanoi Medical University. The questionnaires were developed by the research team and were piloted before being used in the field. The questions included topics such as perception of climate change, impact of climate change, changes in climate as well as illness of family members in summer and winter compared to 5 years earlier.

To characterize the socioeconomic status of the study population, we used principal component analysis to calculate a wealth index based on different variables, including ownership of house, radio, computer, internet connection, telephone, refrigerator, and so on. The wealth index was estimated separately for the non-slum and slum areas, then divided into five quintiles, ranking from the poorest to the richest.

An interview guide based on the broad themes of hot and cold weather and people's perception of climate change was used for the qualitative study. Prior to the official study, a pilot interview was carried out with one female health staff member of the slum area. Interviews were conducted at the interviewees’ house; FGDs were organized in the meeting rooms of two District Health Centers. All of the interviews were recorded both in notes and recordings, with the agreement of the interviewees, and later transcribed.

### Data analysis

Data were analyzed using Stata statistical software, version 12.1. Both descriptive and analytical statistics were applied. The Chi-square test was used to compare discrete variables. Logistic regressions were used to detect any association between people's perceptions of climate change and the impacts of climate change and the social determinants. The independent variables (considered as social determinants) consisted of area of household (slum or non-slum area), education, age group, and socioeconomic status. Significance level was set at *p*<0.05.

Data for qualitative study was transcribed and described according to the above themes of perceptions of climate change and perceptions of impact of climate change on health. Content analysis was used to analyze the interview transcripts. Content analysis is a procedure that organizes transcribed material by coding interview data into blocks that represent a common theme or new themes that emerge from the interviewee quotes (22).

### Ethical considerations

People invited to participate in the study were asked for their informed consent before they were interviewed. They were also informed that they were free to withdraw from the study at any time.

## Results

### The characteristics of the study subjects

Of the 1,444 people selected by the sampling procedure, 1,412 (97.8%) were available and agreed to participate and provided information. Table 1 shows the demographic characteristics of the study population according to living area (non-slum or slum area). The mean age of the respondents was 55.6±16.5 years (range 16–92) in the non-slum areas and 51.3±16.5 (range 16–95) in the slum areas. The distribution of gender, marital status, and wealth index was quite similar between two areas. However, there were significant differences in the distributions of age groups, education, and household size between them. People in the non-slum area seemed to have higher education levels than those in the slum areas, especially more of them had college education.

**Table 1:** Demographic characteristics of study population (n=1,412 people)

	Non-slum area (%)	Slum area (%)	*χ* ^2^	*p*
Gender				
Female	482 (65.8)	436 (64.2)	0.42	0.52
Male	251 (34.2)	243 (35.8)		
Age				
15–24	24 (3.3)	38 (5.6)	32.08	0.00
25–34	76 (10.4)	94 (13.8)		
35–44	101 (13.8)	123 (18.1)		
45–54	104 (14.2)	128 (18.9)		
55–64	195 (26.6)	139 (20.5)		
65–74	144 (19.7)	89 (13.1)		
75+	89 (12.1)	68 (10.0)		
Education				
Primary school or less	50 (6.8)	137 (20.2)	98.90	0.00
Secondary school	158 (21.6)	199 (29.3)		
High school	209 (28.5)	187 (27.5)		
College/university	316 (43.1)	156 (23.0)		
Marital status				
Unmarried	55 (7.5)	62 (9.1)	4.02	0.13
Married	563 (76.8)	490 (72.2)		
Divorced/widowed	115 (15.7)	127 (18.7)		
Household size				
1–3 members	227 (31.0)	329 (48.5)	46.21	0.00
4–6 members	458 (62.5)	309 (45.5)		
≥7 members	48 (6.6)	41 (6.04)		
Socioeconomic status (Wealth index)				
1st (poorest)	145 (19.8)	141 (20.8)	3.29	0.51
2nd	158 (21.6)	124 (18.3)		
3rd	144 (19.7)	142 (20.9)		
4th	154 (21.0)	136 (20.0)		
5th (richest)	132 (18.0)	136 (20.0)		

### Perception of climate change

Most of the survey respondents had heard about climate change and its impact. The proportion of people having heard about climate change and its impact on people was significantly higher in the non-slum area. Among those who had heard about climate change in both slum and non-slum areas, two thirds were aware of the term ‘climate change’ and interpreted it as storms, floods, deep cold, and long heat waves. The impact of climate change most often mentioned by both groups was the impact on human health (92.3 and 91.3% for slum and non-slum areas). However, a small number of respondents were not aware of this term and mentioned other phenomena such as earthquakes or air pollution as manifestations of climate change (13.1 and 12.2% in non-slum and slum areas, respectively) (Table 2).

**Table 2:** Perception of climate change and impacts of climate changes

	Non-slum area (%)	Slum area (%)	*χ* ^2^	*p*
Have you ever heard about climate change? (*n*=1,412)	581 (79.3)	476 (70.1)	15.72	0.00
If yes, what climate change could be? (*n*=1,057)				
Storm	382 (65.8)	325 (68.3)	0.76	0.39
Flood	380 (65.4)	306 (64.3)	0.14	0.71
Deep cold	374 (64.4)	289 (60.7)	1.50	0.22
Drought	274 (47.2)	231 (48.5)	0.20	0.66
Long heat wave	394 (67.8)	302 (63.5)	2.22	0.14
Other (tsunami, earthquake, pollution, hail, etc.)	76 (13.1)	58 (12.2)	0.19	0.66
Have you ever heard about impacts of climate change? (*n*=1,367)	596 (81.3)	470 (69.2)	27.85	0.00
If yes, what are impacts due to climate change? (*n*=1,066)				
Impact on health	544 (91.3)	434 (92.3)	0.39	0.53
Impact on crops	230 (38.6)	166 (35.3)	1.20	0.27
Impact on livestock	202 (33.9)	147 (31.3)	0.82	0.36
Impact on environment	448 (75.2)	324 (68.9)	5.11	0.02
Unknown	14 (2.4)	8 (1.7)	0.54	0.46

This incorrect perception was explained by one man, aged 39 years: ‘I heard from the television that the areas affected by earthquake are often highly vulnerable to climate-related problems, for example more extreme storms’.

Key informant interviews and FGDs revealed that most of the people perceived that ‘climate change is occurring’ in the form of changes in rainfall and temperature; one third perceived it as a change of strong wind during the rainy season. We also asked the participants in a FGD to give their views on the main reason for the changes in climate. Two people said, ‘It is caused mostly by human activities’, while others mentioned that ‘it is caused by both the natural changes in the environment and human activities’.

As shown in Table 3, the majority of the respondents in both non-slum and slum areas stated that changes in climate had occurred during the past 5 years. There were significant differences in perception of climate change in general between those living in non-slum and slum areas. However, there were no significant differences between the non-slum and slum areas when respondents reported about changes in patterns of illness in their families. About one-third of respondents reported that illness among family members had increased during both summer and winter compared to 5 years earlier.

**Table 3:** Perceived changes in heat and cold compared to 5 year ago (*n*=1,412)

	Non-slum area (%)	Slum area (%)	*χ* ^2^	*p*
How do you perceive change in heat in the last summer compared to 5 years ago?				
Hotter	514 (70.1)	464 (68.3)	9.48	0.02
No different	148 (20.2)	116 (17.1)		
Cooler	53 (7.2)	69 (10.2)		
Unknown	18 (2.5)	30 (4.4)		
How do you perceive change in your family members’ sickness in the last summer compared to 5 years ago?				
Sick more	254 (34.6)	260 (38.3)	3.22	0.36
No different	457 (62.4)	394 (58.0)		
Sick less	15 (2.05)	19 (2.8)		
Unknown	7 (0.95)	6 (0.9)		
How do you perceive change in heat in the last winter compared to 5 years ago?				
Colder	453 (61.8)	385 (56.7)	16.67	0.00
No different	156 (21.3)	127 (18.7)		
Warmer	87 (11.9)	134 (19.7)		
Unknown	37 (5.1)	33 (4.9)		
How do you perceive change in your family members’ sickness in the last winter compared to 5 years ago?				
Sick more	256 (43.9)	252 (37.1)	1.52	0.68
No different	447 (61.0)	402 (59.2)		
Sick less	21 (2.9)	20 (2.95)		
Unknown	9 (1.2)	5 (0.74)		

Participants in the qualitative study were also asked to reflect on their perceptions of any changes in illness among their family members compared to 5 years earlier. Most of those interviewed said that their family members, especially children, became ill more easily now than in the past.

One member of health staff said: ‘The number of children coming to our health center has been increasing recently. The hot and humid environment in Hanoi is favorable for the development of bacteria, insects and other disease carriers such as flies and rats. Children are more vulnerable to these diseases’.

According to the respondents, the most common symptoms due to hot weather were headache, fatigue, and dizziness. Hypertension and other cardiovascular diseases were thought to have become more common. The symptoms most often mentioned in relation to cold weather were cough and fever, while common diseases were pneumonia, influenza, and emerging infectious diseases such as dengue fever or Japanese encephalitis.

For example, a male house owner said, ‘Nowadays, there is an increase of infectious diseases such as dengue fever. It occurs periodically throughout the year. The hot weather and rainfall have created favorable conditions for breeding of dengue mosquitoes’.

### Perception of impacts of climate change and social determinants

Table 4 presents the results of the logistic regression analysis of correlates of perception of climate changes impact and selected social determinants. The analysis revealed that for those who lived in both slum and non-slum areas, the perception of climate change impact was significantly associated with gender and education. Men were almost 1.6 times more likely to have heard of the impacts of climate change than women. Respondents in non-slum areas who had completed high school or university were 5.8 and 12.9 times more likely to have heard of climate change than those who had not completed primary school. The odds ratios were similar for the same comparison in slum areas: 4.9 and 11.1. There was no significant association between the perception of impact of climate change and socioeconomic status.

**Table 4:** Association of impacts of climate change perception and social determinants

	Non-slum area (*n*=733)		Slum area (*n*=679)	
	
Independent variables	OR	*P*	OR	*p*
Gender				
Male	1.6	0.04	1.6	0.02
Female	1	–	1	–
Education				
Primary school or below	1	–	1	–
Secondary school	3.5	0.00	1.9	0.00
High school	5.8	0.00	4.9	0.00
College/university	12.9	0.00	11.1	0.00
Socioeconomic status (wealth index)				
1st (poorest)	1	–	1	–
2nd	1.2	0.42	2.7	0.00
3rd	0.9	0.29	1.9	0.02
4th	1.0	0.31	2.7	0.00
5th (richest)	0.6	0.17	3.4	0.00

## Discussion

Most of those living in non-slum areas and more than half living in slum areas had some perception of changes in climate and of the effect on health. According to the majority of respondents, climate change is occurring and results in an increase of temperature during the summer and decrease of temperature during the winter, compared to 5 years ago. They also had the perception that storms, floods, deep cold, and long heat waves were manifestations of climate change. Similar observations have been reported in various reports about climate change from both developed and developing countries, including Japan, Laos, the Philippines, Bangladesh, and several African countries (8, 9, 11, 12, 23). In Japan, in 2008, Aoyagi-Usui compared public opinion surveys from 1997, 2002, 2006, and 2007, and reported that people's awareness of environmental issues was gradually becoming focused on global warming; people were increasingly worried that the rising average temperature of the world would have devastating effects on human life in the next century (11). This phenomenon was also seen in Bangladesh, where the heat during summers was felt to have increased and rainfall to have decreased, compared to 5 or 10 years earlier (12).

Different population groups may have different opinions about climate change (13, 23, 24). We found that those living in non-slum areas and those with higher education levels had heard about climate change significantly more than had people living in slum areas and having less education. Carew-Reid (24) found that poor people seemed to have less knowledge about the adverse impacts of climate change. For example, poor migrants had more difficulties adapting to climate change as a result of having less knowledge, less support and fewer networks, as well as less experience with floods and storms compared to local residents (23). Chelsea and Patrick (25) reported that climate change awareness most strongly depended on the respondent's level of education. Heads of households with higher levels of education were more likely to be aware of climate change than those with lower education levels (26). Another study conducted in Vietnam among poor people suggested that women had less access to information about weather than did men; 60% of the women ‘had not heard’ weather forecast information compared to 35.3% of men, and 35.6% of women had ‘heard but not understood’ the information compared to 26.2% of the men. Limited access to early warning weather information or lack of communications may exacerbate women's difficulties in dealing with climate disaster (24).

The impact of climate change on human health has been seen in several studies worldwide (27, 28). In this study, people reported that they more easily became ill now than some years ago, although most of the symptoms and diseases mentioned were common, with the exception of the increase in emerging diseases such as the new influenzas, dengue and Japanese encephalitis. This perception might be a result of the findings from another study in Northern Vietnam, which showed that a warming climate would change seasonal structure, and a warming winter would result in changes in people's biological rhythm (27). The results of a study conducted in Australia, Canada, UK, USA, and Sweden (28) suggested that a warmer climate could have adverse impacts on people's health which could lead to negative health consequences for vulnerable groups such as old people and those suffering from cardiac diseases, while a colder climate could lead to increases in coughs/colds, headaches, asthma, pneumonia, and especially in re-emerging diseases such as dengue or Japanese encephalitis (28).

A high level of awareness on the links between climate change and human health may help to increase the success of the National Prevention Program on Climate Change. Moreover, understanding the concerns of the people may help policy makers to develop and implement effective and sustainable adaptation measures. However, our results also showed that about 10% of respondents had incorrect ideas, that earthquakes or air pollution were caused by climate change. According to Dr. Nguyen Huu Ninh of the Center for Environment Research Education and Development of Vietnam (personal communication), not many people in Vietnam fully understand about climate change, even among policy makers. Many people are still not aware of the threats of climate change and misunderstand that it is primarily an environmental issue (29).

Re-framing climate change as a threat to human health can be the principle catalyst for people to change their behavior and increase their support for climate change mitigation and adaptation policies. The public will need to learn to appreciate that climate change is a public health issue too, as it affects people's health and well-being.

This study does have some limitations. Firstly, the study was conducted in four urban districts in Hanoi, so it may not represent the perceptions elsewhere in Hanoi or Vietnam. Secondly, the issue under study is complicated and difficult to measure. People's perceptions on changing health could not be checked against real data on health and possible changes related to climate in this study. Such data could be collected in future to give more precision to the accuracy of people's perceptions. There is, however, evidence for an increase in emerging infections such as dengue and Japanese encephalitis in recent years, as shown in another study by Toàn et al. (30).

## Conclusions

Our study revealed that a high proportion of surveyed respondents in the capital city did perceive that climate change is occurring and could mention possible consequences such as heavier rainfall and higher temperatures. The most influential factor on such awareness was the level of education of the respondent; those with higher education had more knowledge about climate change and its impact. Recent climate change has affected Hanoi and may have led to an increase in health problems for its inhabitants.

### Policy recommendations

Understanding local perceptions and concerns will assist policy makers to develop effective communication strategies for each location in Vietnam. In this regard, the main lessons emerging from this study are the following:

- More education and raising awareness is needed for people to appreciate that climate change is a public health issue. A high level of awareness on the links between global environmental change and human health may help to increase the success of the National Prevention Program on Climate Change.
- The Program should make use of community groups as climate change communication channels, especially for the more excluded groups such as women and people with lower levels of education who presently have less awareness of the issues.
